# Case report of portal hepatic schwannoma: presentation of multimodality images

**DOI:** 10.1186/s12876-021-01767-9

**Published:** 2021-04-20

**Authors:** Maki Amano, Yasuo Amano, Ryo Takagi, Xiaoyan Tang, Yuko Omori, Masahiro Okada

**Affiliations:** 1 Department of Radiology, Nihon University Hospital, 1-6 Kanda-Surugadai, Chiyoda-ku, Tokyo, 101-8309 Japan; 2 Department of Pathology, Nihon University Hospital, Tokyo, 101-8309 Japan; 3 Department of Radiology, Nihon University School of Medicine, Tokyo, 173-8610 Japan

**Keywords:** Schwannoma, Portal hepatis, Multimodality imaging, Positron emission tomography

## Abstract

**Background:**

Portal hepatic schwannoma is a rare benign tumor and difficult to diagnose preoperatively because of its rarity and imaging manifestations that mimic malignancy. We present a case of portal hepatic schwannoma that showed moderate contrast enhancement on computed tomography (CT), extension along the bile duct on T2-weighted imaging and magnetic resonance cholangiopancreatography (MRCP), and uptake of ^18^F-fluorodeoxyglucose (FDG) on positron emission tomography.

**Case presentation:**

Ultrasonography at an annual health checkup identified a hepatic mass in a 38-year-old woman. CT showed a well-defined portal hepatic tumor with mild contrast enhancement. T2-weighted imaging and MRCP showed a clavate tumor extending along the intrahepatic bile ducts but no dilatation of the ducts. The tumor exhibited increased FDG uptake, such as maximum standardized uptake values of 5.0 and 6.5 in the early and late phases, respectively. Neither dilatation of intrahepatic bile ducts nor lymphadenopathy was identified, and the multimodality imaging suggested hepatic portal lymphoma, gastrointestinal tumor, or IgG4-related disease rather than cholangiocarcinoma. A needle biopsy via endoscopic ultrasonography was performed, and immunohistology confirmed the tumor as a schwannoma.

**Conclusions:**

The diagnosis of a portal hepatic schwannoma requires immunohistological examinations in addition to multimodality imaging studies to reflect fully the pathohistological characteristics of the tumor.

## Background

Schwannoma is a benign neoplasm that can originate from the inner portion of the peripheral nerve sheath in any body region [1]. Because the periportal region includes autonomic nerve fibers as well as bile ducts, vessels, and lymphatic ducts, schwannomas can occur in this region [2]. However, porta hepatic schwannoma is a rare benign tumor that probably originates from the neural network in the portal hepatis [2–6]. Thus, it is difficult to make an accurate diagnosis of the tumor and differentiate it from cholangiocarcinoma or gastrointestinal tumor (GIST). Computed tomography (CT) and magnetic resonance imaging (MRI) including MR cholangiopancreatography (MRCP) are often performed for the diagnosis of periportal tumors. ^18^F-fluorodeoxyglucose (FDG) positron emission tomography (PET) is commonly used for staging malignant tumors, but FDG can also accumulate in schwannomas of the gastrointestinal systems [7–9]. To our knowledge, only one case report has described the FDG accumulation in the hepatic schwannoma [3].

Herein, we report a case of portal hepatic schwannoma that was examined using multimodality imaging methods. Neither dilatation of intrahepatic bile ducts nor lymphadenopathy was identified, and the multimodality imaging suggested hepatic portal lymphoma, GIST, or IgG4-related disease rather than cholangiocarcinoma. Immunohistological examinations of tissue sampled by needle biopsy confirmed the diagnosis of schwannoma.

## Case presentation

**Fig. 1:**
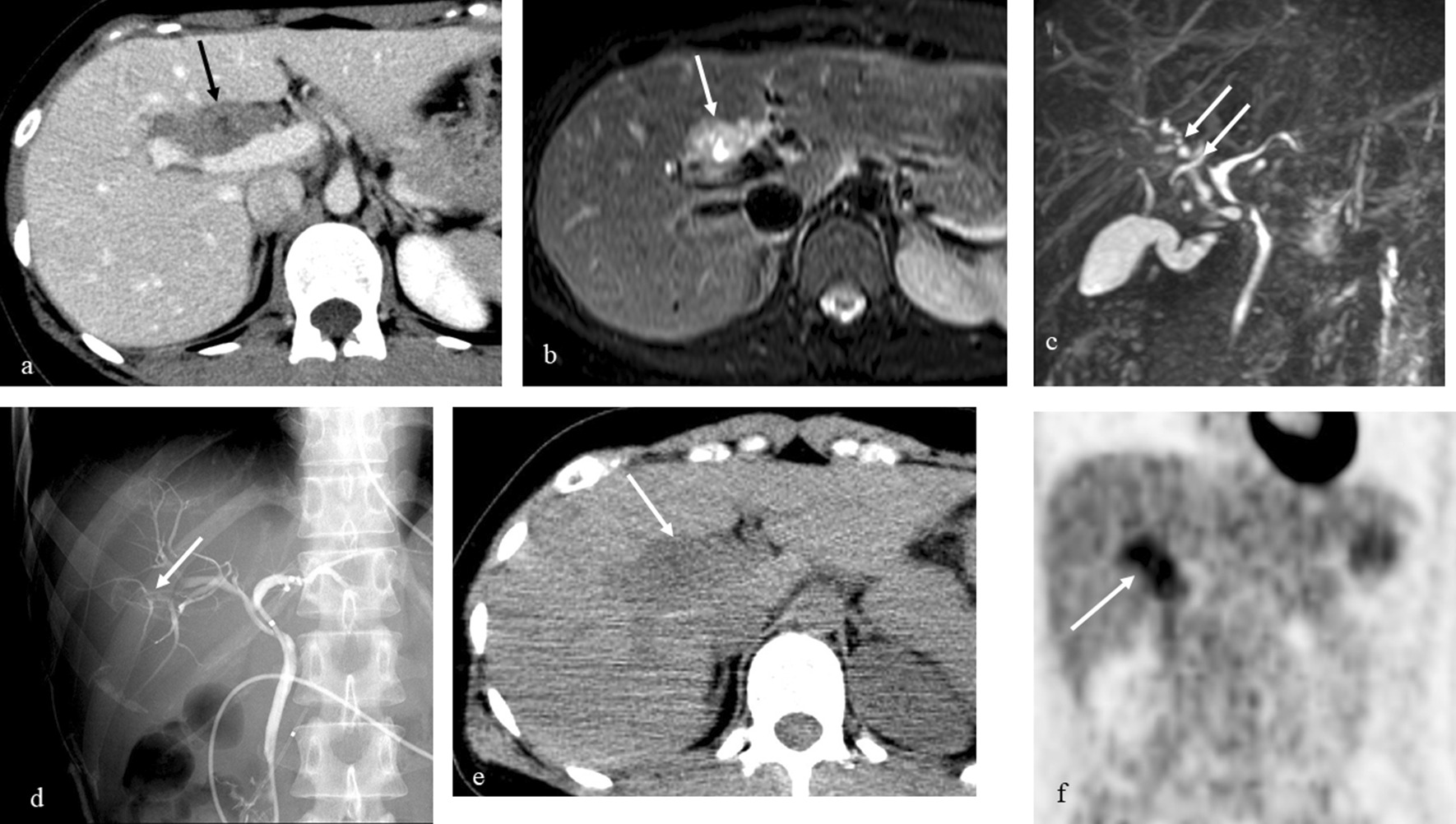
**a** Contrast-enhanced CT shows a clavate-shaped periportal tumor with inhomogeneous moderate enhancement (arrow). **b** T2-weighted imaging shows the tumor isointense to fat with cystic degeneration (arrow). **c** MRCP shows bile duct compression without any biliary dilatation (arrows). **d** ERCP also shows bile duct compression without any biliary obstruction (arrow). **e** The tumor has not changed at the 2nd CT (arrow). **f** PET shows FDG uptake of the tumor (arrow, SUV_max_ 5.0). SUV_max_ is increased slightly to 6.5 in the late phase

**Fig. 2:**
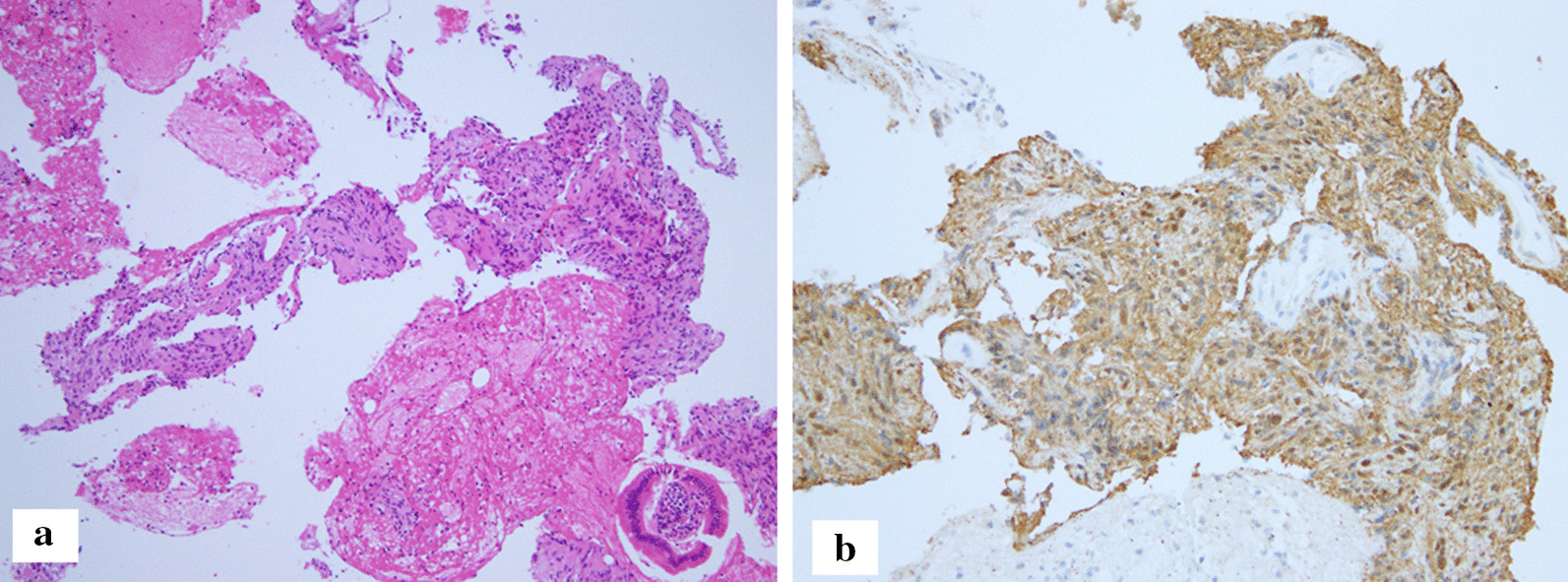
Histological findings: **a** proliferation of spindle tumor cells (hematoxylin and eosin staining). **b** The tumor cells are strongly positive for S-100

A 38-year-old woman underwent an annual health checkup, and the ultrasonography identified a hepatic mass close to the portal vein. No specific findings were noted in her personal and family history. Her physical and laboratory data were unremarkable. CT showed a well-defined portal hepatic tumor with inhomogeneous moderate contrast enhancement (Fig. 1a). The tumor measured 4.2 cm × 6.8 cm. The clavate tumor showed low intensity on T1-weighted imaging and was isointense to fat on T2-weighted imaging (Fig. 1b). The tumor included cystic degeneration and extended along the intrahepatic bile duct (Fig. 1b). Both MRCP and endoscopic retrograde cholangiopancreatography showed focal bile duct compression without any biliary obstruction and dilatation (Fig. 1c, d). ^18^F-FDG accumulation was shown on PET after 5 months of the CT scan; the size of the tumor did not change during the interval (Fig. 1e), and the maximum standardized uptake value (SUV_max_) was 5.0 (Fig. 1f) and 6.5 in the early and late phases, respectively. Lymphadenopathy was not identified by any imaging methods. Because IgG4-related disease and portal hepatic malignancy, including malignant lymphoma and cholangiocarcinoma, were still of our concern, a needle biopsy via endoscopic ultrasonography was performed. Histological study showed the spindle cells close to the bile duct (Fig. 2a). The lymphocyte infiltration was not identified, but the small vessels had proliferated moderately. Immunohistological examinations showed that the tumor cells were strongly positive for S-100 (Fig. 2b) but not for smooth muscle actin and c-Kit. Ki-67 staining was < 1% of the tumor cells. The diagnosis of portal hepatic schwannoma was made. Because of her age and possibly high invasiveness of the surgery for the portal benign tumor, we did not recommend the surgical procedures. She was followed up intensely in another institution.

## Discussion and conclusions

Schwannoma can originate from the inner portion of any peripheral nerve sheath except the olfactory and optic nerves, which lack Schwann cells [1]. Portal hepatic schwannoma is rare, despite the distribution of autonomic neural networks along the hepatic arteries. Yin et al. [6] reported that the median size of the tumor is 4.7 cm and 40% of patients are asymptomatic, and our findings were consistent with these values. The physical and laboratory data for our patient were unremarkable; neither superficial lymphadenopathy nor jaundice was observed. Associated neurofibromatosis was not identified. Our findings suggest that multimodality imaging should be performed to evaluate the portal hepatic tumor before surgery.

Manifestations shown with multimodality imaging may reflect some pathohistological characteristics of a portal hepatic schwannoma. The clavate shape of the tumor can be consistent with the tumor distribution along the neural networks. The inhomogeneous contrast enhancement on CT and the presence of cystic degeneration on T2-weighted images suggested the coexistence of areas rich in spindle cells (Antoni A) and hypocellular areas (Antoni B) within the neurogenic tumor [5, 6]. Compared to other types of nerve sheath tumors such as neurofibroma and melanotic schwannoma, the present MRI findings were consistent with schwannoma [10]. T2-weighted and MRCP images showed that the tumor extended along the bile ducts but no dilatation of the peripheral bile ducts. This imaging finding was not typical for cholangiocarcinoma. As reported about gastrointestinal schwannoma [7–9], FDG accumulated in the portal hepatic schwannoma in our patient. Because Ki-67 staining was very low (< 1%) and lymphoid cuff was not identified histologically, neither malignant potential nor reactive inflammation was the cause of the FDG accumulation in the tumor. The hypercellularity and microvascular density may be related to the FDG accumulation in the portal hepatic schwannoma. The glucose transporter might facilitate the uptake of FDG in schwannomas [9].

It was difficult to diagnose portal hepatic schwannoma and differentiate it from malignant lymphoma, GIST, or IgG4-related disease. Although the SUV_max_ of 5.0 was low for a typical malignant lymphoma, the FDG avidity of a lymphoma can vary between the histological types [11]. GIST and IgG4-related disease can affect the periportal region and show the uptake of FDG on PET [12, 13]. Because multimodality imaging indicated a low possibility of cholangiocarcinoma, we performed a needle biopsy, but not surgical intervention for this patient because of her age and high invasiveness of the hepatic surgery. Malignant schwannoma has been reported as the portal hepatic neurogenic tumor with high mitosis [14]; the tumor is a progressive and mostly cystic lesion, and involves the hepatic parenchyma or bile ducts. Some cases of the malignant schwannoma are associated with neurofibromatosis. These clinical and imaging features may be different from those of our case. Although the accurate diagnosis of portal hepatic schwannoma remains difficult, multimodality imaging allowed us to use a less invasive procedure to acquire the histological specimen.

In conclusion, this was a rare case of portal hepatic schwannoma that showed FDG accumulation on PET. Several imaging characteristics suggested that this was a benign tumor in the periportal region. The diagnosis of portal hepatic schwannoma requires immunohistological examinations in addition to multimodality imaging studies to reflect fully the pathohistological characteristics of the tumor.

## Data Availability

The clinical and imaging data are available from the corresponding author upon request.

## Abbreviations

CT: Computer tomography
FDG: Fluorodeoxyglucose
GIST: Gastrointestinal tumor
MRI: Magnetic resonance imaging
MRCP: MR cholangiopancreatography
PET: Positron emission tomography
SUV_max_: Maximum standardized uptake value
